# Modifiable risk factors associated with bone deficits in childhood cancer survivors

**DOI:** 10.1186/1471-2431-12-40

**Published:** 2012-03-28

**Authors:** Lynda E Polgreen, Anna Petryk, Andrew C Dietz, Alan R Sinaiko, Wendy Leisenring, Pam Goodman, Lyn M Steffen, Joanna L Perkins, Donald R Dengel, K Scott Baker, Julia Steinberger

**Affiliations:** 1 Department of Pediatrics, University of Minnesota, Minneapolis, MN, USA; 2 Department of Pediatrics, University of California San Diego and Rady Children's Hospital, San Diego, CA, USA; 3 Clinical Research Division, Fred Hutchinson Cancer Research Center, Seattle, WA, USA; 4 School of Public Health, University of Minnesota, Minneapolis, MN, USA; 5 Children's Hospitals & Clinics of Minnesota, Minneapolis, MN, USA; 6 School of Kinesiology, University of Minnesota, Minneapolis, MN, USA; 7 Pediatric Endocrinology, University of Minnesota, East Building Room MB671 2450 Riverside Ave., Minneapolis, MN 55454, USA

## Abstract

**Background:**

To determine the prevalence and severity of bone deficits in a cohort of childhood cancer survivors (CCS) compared to a healthy sibling control group, and the modifiable factors associated with bone deficits in CCS.

**Methods:**

Cross-sectional study of bone health in 319 CCS and 208 healthy sibling controls. Bone mineral density (BMD) was measured by dual-energy x-ray absorptiometry (DXA). Generalized estimating equations were used to compare measures between CCS and controls. Among CCS, multivariable logistic regression was used to evaluate odds ratios for BMD Z-score ≤ -1.

**Results:**

All subjects were younger than 18 years of age. Average time since treatment was 10.1 years (range 4.3 - 17.8 years). CCS were 3.3 times more likely to have whole body BMD Z-score ≤ -1 than controls (95% CI: 1.4-7.8; p = 0.007) and 1.7 times more likely to have lumbar spine BMD Z-score ≤ -1 than controls (95% CI: 1.0-2.7; p = 0.03). Among CCS, hypogonadism, lower lean body mass, higher daily television/computer screen time, lower physical activity, and higher inflammatory marker IL-6, increased the odds of having a BMD Z-score ≤ -1.

**Conclusions:**

CCS, less than 18 years of age, have bone deficits compared to a healthy control group. Sedentary lifestyle and inflammation may play a role in bone deficits in CCS. Counseling CCS and their caretakers on decreasing television/computer screen time and increasing activity may improve bone health.

## Introduction

Osteoporosis is a systemic skeletal disease characterized by low bone mass and microarchitectural deterioration, resulting in an increased susceptibility to fracture [1]. Reduced bone mineral density (BMD) is a recognized condition among childhood cancer survivors (CCS). It is estimated that up to 46% of CCS less than 18 years old have reduced BMD [2-8]. Although children usually recover from fractures without any complication, fractures in adults have been shown to significantly increase both morbidity and mortality [9,10]. Importantly, the majority of bone accretion occurs in adolescence and young adulthood with peak bone mass reached by the second or third decade [11]. Treatment during adolescence interrupts this critical period of bone acquisition. A resultant decrease in peak bone mass would be expected to increase the risk of osteoporosis and osteoporotic fractures later in life [12].

The known risk factors for reduced BMD in CCS include treatment with glucocorticoids [6,13-16], radiation [16-21], methotrexate [2,6,16,18,21], and endocrine insufficiencies such as growth hormone (GH) deficiency [5,17,22] and hypogonadism [6,17,23] that are sequelae of cancer treatment. While efforts are made to limit the exposure to these agents without compromising their effectiveness, there are limitations to these approaches due to the nature of the disease and available treatment options. Hence, there is a need to identify modifiable risk factors to design appropriate preventive and therapeutic interventions during childhood and adolescence while there is still potential for BMD gain. Currently there are limited data on modifiable lifestyle factors that could influence bone health in CCS, therefore we undertook a study to evaluate the associations between potentially modifiable factors and bone deficits in CCS. We hypothesized that CCS will have lower BMD compared to the sibling control group and that low activity, low lean body mass, high percent body fat, higher levels of markers of inflammation, and lower dietary calcium, vitamin D and zinc intake will be associated with bone deficits in CCS. These data could be used to focus bone health promoting interventions and by pediatricians during routine health maintenance visits to guide counseling of CCS on ways to improve bone health, prevent osteoporosis and reduce the risk of fractures.

## Methods

The study was approved by the Institutional Review Board: Human Subjects Committee at the University of Minnesota Medical Center and Children's Hospitals and Clinics of Minnesota. Consent (and assent as appropriate) was obtained from children and their parent/guardian(s). We identified 723 living subjects, ages 9-18 years old, treated for cancer at the University of Minnesota Amplatz Children's Hospital and the Children's Hospitals & Clinics of Minnesota, in remission and surviving for ≥ 5 years after diagnosis of leukemia, central nervous system (CNS) tumors and solid tumors. Of these, 66 were not able to be contacted; of the remaining 657, 319 (49%) agreed to participate. 110 had leukemia, 127 solid tumors (i.e. sarcoma, renal, neuroblastoma, non-Hodgkin's lymphoma), and 82 CNS tumors (i.e. glial tumors, retinoblastoma, neuroectodermal tumors). 134 had a history of corticosteroid treatment, and 74 had a history of treatment with radiation (31 cranial radiation). Participants treated with hematopoietic cell transplantation (HCT) were excluded. There were no significant differences in age, sex, race, diagnosis, age at diagnosis and length of follow-up (time from diagnosis to study evaluation) between CCS participants and non-participants. A contemporary control group of 208 healthy siblings of CCS were recruited. Controls known to suffer from chronic illnesses including hypothyroidism and delayed puberty, or at risk for GH deficiency (i.e. height > 2 standard deviation (SD) below the mean and height velocity > 2 SD below the mean) were excluded from participation.

Following a 10-12 hour overnight fast, all participants underwent a physical examination (including Tanner staging [24] by a trained study physician), height measured by wall mounted stadiometer (without shoes) to the nearest 0.1 cm and weight by electronic scale to the nearest 0.1 kg, and laboratory testing, including free thyroxine (free T4) by competitive immunoassay (CV 5.8-7.3%), thyroid stimulating hormone (TSH) (CV 4.9%), follicle stimulating hormone (FSH) (CV 5.8-6.1%), and insulin-like growth factor-1 (IGF-1) (CV 5.2-6.4%) by chemiluminescent immunoassay (Siemens Healthcare Diagnostics, Tarrytown, NY); and interleukin-6 (IL-6) (CV 14.5%) by ELISA (R&D Systems, Minneapolis, MN), adiponectin (CV 17.1%) and leptin (CV 13.7%) by 2-plex competitive immunoassay on the Luminex platform (Austin, TX) using bead sets from R&D Systems (Minneapolis, MN). GH stimulation test using clonidine and arginine was performed. GH deficiency was defined as a stimulated peak GH level less than 7 mcg/L, which is a more conservative cutoff than 10 mcg/L, which is frequently used in clinical practice to diagnose GH deficiency [25]. Total body (not excluding head) BMD, posterior anterior lumbar spine (L2-L4), and body composition (percent body fat and lean body mass) were assessed by dual-energy x-ray absorptiometry (DXA) (G.E. Lunar Prodigy scanner; pediatric software version 9.3; Madison, WI, USA), and bone age by the Greulich and Pyle method [26]. Dietary intake was evaluated using the Youth/Adolescent Questionnaire (YAQ) [27,28]. Physical activity, including television/computer screen time, was assessed by the Modifiable Activity Questionnaire for Adolescents [29]. The diagnoses of hypogonadism was made by either participant report of a history of diagnosed hypogonadism or for males an LH > 10 IU/L and testosterone below the lower end of reference range for pubertal stage and for females an FSH > 40 IU/L. The diagnosis of hypothyroidism was made by participant report, high TSH (> 5.0 uU/mL) and normal free T4, or low free T4 (< 0.8 ng/dL). Height and weight Z-scores were calculated based on 2000 Centers for Disease Control growth charts.

According to current International Society for Clinical Densitometry (ISCD) recommendation the terms osteoporosis should be limited to children with low BMD (Z-score ≤ -2) accompanied by fractures [30]. Since very few patients met the definition of osteoporosis or low BMD in our study, yet a substantial proportion of patients had BMD below average, we defined a mild BMD deficit as a Z-score ≤ -1. This cutoff has been used in other studies describing bone deficits in CCS [22,31]. The rationale for using this cutoff is that those with lower BMD Z-scores are likely to remain in the low end of the normal range [32] and reach a lower peak BMD [11] thus increasing their lifetime risk of osteoporosis and fracture. In addition, any decrease in BMD Z- score predisposed children to an increased risk of fracture [33,34].

### Statistical analysis

Descriptive statistics are expressed as frequencies and percents or mean ± standard error (SE), as appropriate. Regression models based on generalized estimating equations (GEE) with robust variance estimates were used to compare measures between CCS and the sibling control group with adjustments as noted in tables, to appropriately account for intra-family correlation. Among CCS, multivariable logistic regression was used to evaluate odds ratios (OR) for the associations between Z-score ≤ -1 of the whole body BMD and lumbar spine BMD with the following predictors: age at diagnosis, time since diagnosis, GH Status (GH deficient, not GH deficient), IGF-1 SDS (≤ -2, > - 2), percent body fat, lean body mass, body mass index (BMI), IL-6, adiponectin, leptin, calcium intake, vitamin D intake, zinc intake, omega-3 intake, protein intake, milk intake, fruit and vegetable intake, physical activity score, television/computer screen time, time elapsed since diagnosis, radiation and steroid exposure. All models were adjusted for sex, age-at-study, ethnicity (white-not-Hispanic, others), and pubertal Tanner stage.

The assumption of linearity for continuously valued factors was evaluated using Generalized Additive Models (GAM) [35], and relevant categorical variables were created for those that were significantly non-linear at the alpha = 0.05 level, and, for adiponectin, leptin and IGF-1 to minimize undue influence by extremely large values [35]. Age at study (whole body analysis only), IL-6, time elapsed since diagnosis (lumbar spine analysis only), and calcium were categorized per non-linear relationship with the outcomes. Category cut points were selected based on visual inspection of predicted curves from GAM models, and practical considerations regarding numbers of events per category. Because of correlation between percent body fat and leptin in both the whole body and lumbar spine models, separate multivariable models were developed including either leptin or percent body fat. Because of correlation between hypogonadism and hypothyroidism in the lumbar spine model, separate multivariable models were developed including either hypothyroidism or hypogonadism. Five models were evaluated: model 1: IGF-1 SDS, hypothyroidism and/or hypogonadism; model 2: lean body mass, percent fat mass (or leptin), television/computer screen time, physical activity score, years since diagnosis and IL-6; model 3: milk, protein, fruits/vegetable and daily total caloric intakes; model 4: protein, vitamin D, zinc, calcium, omega-3 and daily total caloric intakes; model 5: radiation and steroid exposure. All models were adjusted for age at study, sex, pubertal Tanner stage, and ethnicity. All p-values are two-sided and those < 0.05 were considered statistically significant, and those between 0.05 and 0.10 suggestive of association.

## Results

### Participant characteristics

We evaluated 527 participants, 319 childhood cancer survivors (148 females) aged 9-18 years and 208 controls (97 females) aged 9-18 years (Table 1). CCS were slightly older than controls, however pubertal Tanner stage was similar between groups. CCS were generally of normal stature, but were shorter than controls. Percent body fat was higher and lean body mass lower in CCS, and there were significantly more CCS with obesity (BMI ≥ 95%) than controls.

**Table 1:** Population characteristics of childhood cancer survivors (CCS) compared to controls

		CCS(n = 319)	Controls(n = 208)	
		Mean ± SE	Mean ± SE	*P*
Age at Study, years		14.5 ± 0.1	13.7 ± 0.2	< 0.001
Height, Z-score		0.1 ± 0.1	0.4 ± 0.1	< 0.001
Weight, Z-score		0.5 ± 0.1	0.6 ± 0.1	0.363
% Body Fat^†^		28.1 ± 0.8	25.9 ± 0.9	0.007
Fat Mass, kg^†^		16.5 ± 0.7	14.8 ± 0.8	0.024
Lean Body Mass, kg^†^		38.5 ± 0.5	39.8 ± 0.6	0.015
IGF-1 SDS		-1.24 ± 0.05	-1.04 ± 0.06	0.004
IL-6, pg/ml^†^		1.4 ± 0.1	1.6 ± 0.3	0.505
Bone Age, years		14.5 ± 0.1	13.8 ± 0.2	< 0.001
Relative Bone Age		1.01 ± 0.01	1.01 ± 0.01	0.425
Tanner Score		3.6 ± 0.1	3.3 ± 0.1	0.068
Calcium Intake, mg/day		1280 ± 30	1257 ± 38	0.575
Vitamin D Intake, IU/day		331 ± 10	330 ± 12	0.968
Physical Activity Score, MET - minutes per week		58.6 ± 3.8	65.9 ± 4.9	0.191
		**N (Percent)**	**N (Percent)**	
Screen time	> 1 hr/day	225 (75.5)	147 (73.9)	0.680
	0-1 hr/day	73 (24.5)	52 (26.1)	
Ethnicity	White/not Hispanic	274 (85.9)	194 (93.3)	< 0.001
	Others	45 (14.1)	14 (6.7)	
Sex	Males	171 (53.6)	111 (53.4)	0.956
	Females	148(46.4)	97 (46.6)	
BMI	< 5%	9 (2.8)	4 (1.9)	0.495
	≥ 5% to < 85%	210 (65.8)	137 (65.9)	0.995
	≥ 85% to < 95%	42 (13.2)	43 (20.7)	0.031
	≥ 95%	58 (18.2)	24 (11.5)	0.028

^†^Adjusted for age, sex, ethnicity, and pubertal Tanner stage

Relative Bone age = Bone age/actual age; MET = metabolic equivalent

By history or laboratory assessment on day of study, 2 CCS had type 1 diabetes mellitus, 2 had type 2 diabetes mellitus, 31 had hypothyroidism (all on treatment with normal free T4 levels on day of study), 22 had hypogonadism, 4 had precocious puberty and 16 (5%) had short stature (height less than 3^rd ^percentile for age and gender). Thirty-six (13%) CCS were diagnosed with GH deficiency by stimulation testing at the time of the study and 34 (11%) were diagnosed prior to study entry. For CCS, the average age at cancer diagnosis was 4.5 ± 0.2 years (range 0 - 12.5 years), and the average time since treatment was 10.1 ± 0.2 years (range 4.3 - 17.8 years). The median radiation dose in 31 CCS participants treated with CNS radiation was 2370 cGY (range 1800-5580 cGY). The median number of days of steroid treatment in 133 CCS treated with steroids was 162 days (range 4-302 days) and the median dose 7,520 mg/kg/day prednisone equivalents (range 200-15,250 mg/kg/day prednisone equivalents).

### Bone mineral density

**Figure 1:**
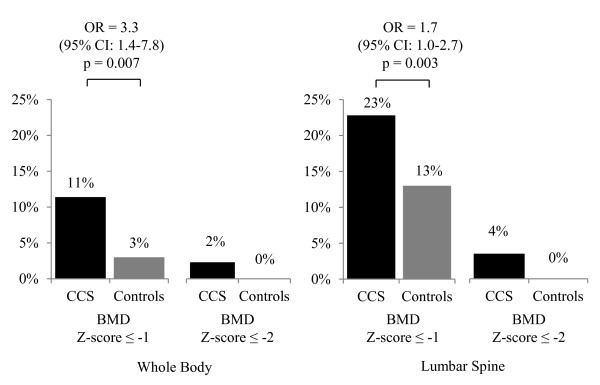
**Whole body and lumbar spine bone deficits in childhood cancer survivors (CCS) compared to controls**. Odds ratio (OR) adjusted for sex, age-at-study, ethnicity (white-not-Hispanic, others), and pubertal Tanner stage are presented for BMD Z-score ≤ -1; OR for BMD Z-score ≤ -2 unable to be calculated due to 0% prevalence in controls.

CCS were significantly more likely to have both whole body and lumbar spine BMD Z-score ≤ 1 (Figure 1) as compared to controls. The mean whole body BMD Z-score and lumbar spine BMD Z-score were lower in CCS vs. controls (unadjusted mean ± SEM: 0.3 ± 0.07 CCS vs. 0.6 ± 0.07 controls; p = 0.002, and -0.2 ± 0.06 CCS vs. 0.1 ± 0.07 controls; p = 0.02 respectively); however, after adjusting for height SDS there was no difference in whole body or lumbar spine BMD Z-scores. There were very few CCS (whole body N = 7 (2.3%), lumbar N = 11 (3.5%)) or controls (whole body = 0, lumbar = 0) with BMD Z-scores ≤ -2. There was no evidence of differential time-since diagnosis effects between subjects who were < 10 years compared to those ≥ 10 years at diagnosis.

### Factors associated with reduced BMD in childhood cancer survivors

In multivariable analysis, testing age, gender, ethnicity, pubertal Tanner stage, IGF-1 SDS and hypothyroidism and/or hypogonadism (model 1), CCS with hypogonadism were 9.1 times more likely to have whole body BMD Z-score ≤ -1 (95% CI: 3.3-25.3; p < 0.001) and 4.4 times more likely to have lumbar spine BMD Z-score ≤ -1 (95% CI: 1.7-11.4; p = 0.002). CCS with hypothyroidism were 2.9 times more likely to have lumbar spine BMD Z-score ≤ -1 (95% CI: 1.3-6.6; p = 0.012). Both hypogonadism and hypothyroidism can cause short stature. Therefore, we added height SDS to the modeling and found similar results. CCS with hypogonadism were 11.2 times more likely to have whole body BMD Z-score ≤ -1 (95% CI: 3.7-35.8; p < 0.001) and 4.3 times more likely to have lumbar spine BMD Z-score ≤ -1 (95% CI: 1.6-11.8; p = 0.003). CCS with hypothyroidism were 2.8 times more likely to have lumbar spine BMD Z-score ≤ -1 (95% CI: 1.2-6.7; p = 0.017). Neither GHD nor low IGF-1 SDS increased the odds of whole body or lumbar spine BMD Z-score ≤ -1. We were next interested in potential effects of a sedentary lifestyle (obesity and low lean body mass) and hormones influenced by adiposity (IL-6, adiponectin, leptin) on bone density. Given the influence of GH deficiency on lean body mass we evaluated the association between lean body mass and GH deficiency; as there was no significant association (p = 0.12) we did not include GH deficiency in these models. In whole body multivariate modeling of lean body mass, percent fat mass (or leptin), television/computer screen time, physical activity score, years since diagnosis and IL-6 (model 2), CCS with higher IL-6 (> 2.5 pg/ml), two or more hours of television/computer screen time per day, and lower lean body mass, were more likely to have whole body BMD Z-score ≤ -1. Percent body fat, adiponectin, years since diagnosis, and physical activity score were not significantly associated with whole body BMD Z-score ≤ -1 (Tables 2 and 3). The addition of height SDS to this model did not change theses associations and did not independently influence the odds of BMD Z-score ≤ -1. In contrast to the results for whole body BMD, in lumbar spine multivariate modeling, IL-6 and screen time were not associated with BMD. However, even after adjusting for age, sex, and pubertal stage, both decreases in physical activity score and lower lean body mass (≤ 35 kg) were significantly associated with the odds of lumbar spine BMD Z-score ≤ -1; after the addition of height SDS to this model, the odds ratio for lean body mass was attenuated (OR 2.9, 95%CI: 1.2-7.4; p = 0.02) although height SDS did not independently influence the odds of lumbar spine BMD Z-score ≤ -1.

**Table 2:** Multivariable analysis in CCS of sedentary lifestyle (obesity and low lean body mass) and hormones influenced by adiposity (IL-6 and adiponectin) associated with BMD Z-score ≤ -1

Outcome: Whole Body BMD Z-score ≤ -1				
	Categories	OddsRatio	95%CI	*P*
Age at study	≤ 16 years	1.0	**-**	**-**
	> 16 years	2.5	0.8-8.1	0.125
Ethnicity	Other	1.0	-	-
	White non-Hispanic	1.7	0.5-6.9	0.420
**Tanner stage, 1-5**	One stage increase	2.2	1.3-3.8	**0.006**
Sex	Female	1.0	-	-
	Male	2.6	0.8-10.0	0.137
Percent Body Fat	1% increase	0.95	0.9-1.0	0.070
**Lean Body Mass**	1 kg increase	0.85	0.8-0.9	**< 0.001**
Years Since Diagnosis	5-9 years	1.0	-	-
	> 9.0 years	2.3	0.9-6.3	0.080
**IL-6**	≤ 2.5 ng/dl	1.0	-	-
	> 2.5 ng/dl	4.4	1.5-12.9	**0.007**
Screen time*, per day	0-1 hours	1.0	-	-
	≥ 2 hours	4.1	1.3-18.6	**0.033**

Physical activity score and adiponectin were not significant factor s (p-value > 0.1) and were therefore removed from the model

*Screen time = television and computer screen time

**Table 3:** Multivariable analysis in CCS of sedentary lifestyle (obesity and low lean body mass) and hormones influenced by adiposity (IL-6 and adiponectin) associated with BMD Z-score ≤ -1

Outcome: Lumbar spine BMD Z-score ≤ -1				
	Categories	OddsRatio	95%CI	*P*
**Age at Study**	1 year increase	2.2	1.7-3.0	**< 0.001**
Ethnicity	Other	1.0	-	-
	White non-Hispanic	2.6	0.9-9.1	0.104
**Tanner, 1-5**	One stage increase	0.4	0.2-0.7	**< 0.001**
Sex	Female	1.0	-	-
	Male	1.6	0.7-3.4	0.270
**Lean Body Mass**	> 35 kg	1.0	-	-
	≤ 35 kg	4.1	1.8-9.6	**< 0.001**
Percent Body Fat	1% increase	0.97	0.94-1.0	0.088
**Physical Activity Score**	One unit increase	0.99	0.99-1.0	**0.042**

Years since diagnosis, IL-6, adiponectin, and screen time were not significant factors (p-value > 0.1) and were therefore removed from the model

Percent body fat and years since diagnosis were not associated with lumbar spine BMD Z-score ≤ -1 (Tables 2 and 3). Leptin and percent body fat were strongly correlated; therefore, two models were built, one that excluded leptin, and one that excluded percent body fat. Similarly, when leptin was substituted for percent body fat, it was not significantly associated with whole body or lumbar spine BMD.

Of all the dietary and nutrient factors (model 3: milk, protein, fruits/vegetable and daily total caloric intake and model 4: protein, vitamin D, zinc, calcium, omega-3 and daily total caloric intake), only total protein was a marginally significant predictor of whole body BMD Z-score ≤ -1 (p = 0.055): the odds of whole body BMD Z-score ≤ -1 were 3% lower for each additional 1 gram of total protein intake. There were no significant associations in model 4.

Finally, the odds of whole body or lumbar spine BMD Z-score ≤ -1 were significantly higher for both those with CNS radiation and with other radiation, as compared to those with no radiation (Tables 4 and 5); however when height SDS was added to the model the odds of lumbar spine BMD Z-score ≤ -1 (but not for whole body) were significantly attenuated for those who received CNS or other radiation. The odds of lumbar BMD Z-score ≤ -1 for those exposed to steroids were almost twice that for those who did not receive steroids (Tables 4 and 5); when height SDS was added to the model the odds were significantly attenuated. The association between whole body BMD Z-score ≤ -1 and steroids was not significant with or without height SDS in the model. Subjects with a higher steroid exposure, a more recent history of steroid exposure or a longer duration of steroid exposure did not show larger BMD deficits.

**Table 4:** Multivariable analysis of cancer treatment factors associated with BMD Z-score ≤ -1

**Outcome: Whole body BMD Z-score ≤ -1**.				
	Category	OddsRatio	95%CI	*P*
Age at Study	≤ 16 years	1.0	-	-
	> 16 years	2.0	0.8-5.3	0.177
Ethnicity	Other	1.0	-	-
	White non-Hispanic	0.9	0.3-2.9	0.800
Tanner stage, 1-5	One stage increase	0.9	0.6-1.4	0.666
Sex	Females	1.0	-	-
	Males	1.2	0.5-2.5	0.704
**Radiation Exposure**	None	1.0	-	-
	CNS Radiation	7.9	3.0-20.8	**< 0.001**
	Other Radiation	5.7	2.3-13.9	**< 0.001**

Steroid exposure was not a significant factor (p-value > 0.1) and was therefore removed from the model

**Table 5:** Multivariable analysis of cancer treatment factors associated with BMD Z-score ≤ -1

**Outcome: Lumbar spine BMD Z-score ≤ -1**.				
	Category	OddsRatio	95%CI	*P*
**Age at Study**	≤ 16 years	1.0	-	-
	> 16 years	3.1	1.4-7.1	**0.006**
Ethnicity	Other	1.0	-	-
	White non-Hispanic	2.2	0.9-6.7	0.135
**Tanner stage, 1-5**	One stage increase	0.7	0.5-0.9	**0.015**
Sex	Female	1.0	-	-
	Male	1.1	0.6-1.9	0.732
**Radiation Exposure**	None	1.0	-	-
	Cranial Radiation	2.5	1.0-5.7	**0.040**
	Other Radiation	2.4	1.0-5.5	**0.041**
**Steroid Exposure**	No	1.0	-	-
	Yes	1.9	1.0-3.5	**0.042**

## Discussion

In this study of childhood cancer survivors (CCS) < 18 years of age whole body and lumbar spine BMD deficiencies (BMD Z-scores ≤ -1) were significantly more common than in a healthy sibling control group. This is important for lifetime bone health, because children with BMD Z-scores ≤ -1 are likely to have a lower peak bone density that sets them up for early osteoporosis and increased risk for fracture later in life. In addition, even during childhood, lower BMD Z-scores are associated with increased risk of fracture [33,34,36]. Although we have no longitudinal data, we found no cross-sectional evidence to suggest "catch-up" in BMD for participants treated at a younger, pre-pubertal, age (< 10 years) and confirmed that hypogonadism is associated with bone deficits in CCS. Finally, we identified modifiable factors associated with lower BMD in CCS: lower lean body mass (independent of GH deficiency status), higher daily television/computer screen, lower physical activity score, and higher IL-6. These risk factors may be targets for medical and lifestyle interventions to increase bone density and decrease the risk of early osteoporosis and fracture in children with CCS.

We found that lower lean body mass increased the likelihood of having a BMD Z-score ≤ -1 independent of the influence of GH deficiency which was not associated with lean body mass. CCS had significantly lower lean body mass than controls; clinically we suspected this was at least in part due to decreased physical activity in CCS; however we did not find a difference in physical activity score between CCS and controls. This may be due to the limitations of quantifying physical activity by questionnaire. Other studies in healthy pediatric populations have shown that increased muscle mass is associated with increased bone mineral content, density, and estimated bone strength [37-40] and that lean body mass, along with male gender and physical activity, can explain up to 37% of total variance in BMD in healthy, prepubertal children [41]. Therefore, these reports suggest that interventions aimed at increasing lean body mass may improve bone density and strength in CCS.

Although physical activity was no different between CCS and controls, lower physical activity in CCS increased the likelihood of having a BMD Z-score ≤ -1. The long-term beneficial impact of increased physical activity on bone was demonstrated in a twin study showing that twins with higher levels of leisure time physical activity for at least 30 years had higher estimated bending and compression strength of bone [42]. Increased physical activity has also been shown to improve bone health in healthy children [43]. Studies on the relationship between physical activity and measures of bone health in CCS have reported either positive associations, similar to our data [2,6,15,32], or no associations [4,7,21,44]. Some of the variability in results is likely related to limitations of activity questionnaires to accurately measure activity level and/or intensity. Adding accelerometers to questionnaires to measure physical activity in CCS uncovered a positive correlation between activity levels and both whole body BMD and estimated volumetric lumbar spine BMD [2] and may provide a more accurate assessment of daily activity level.

Television/computer screen time is used as a surrogate measure of physical activity [45]. The present cohort was divided between CCS who watched television/computer screen for ≥ 2 hours per day versus < 2 hours per day; the CCS with the higher television/computer screen were more likely to have whole body BMD Z-scores ≤ -1. This is similar to a prior report that time spent on television and computers negatively correlated with BMD Z-scores in CCS [5]. In contrast, no association between viewing time and bone measures were found in another study of CCS [3]. The equivocal findings again may be due to limitations in using television viewing time as a surrogate measure for activity. In addition, intensity of exercise is not accounted for when simply quantifying television/computer screen time.

Higher levels of the inflammatory cytokine IL-6 were associated with a BMD Z-score ≤ -1 in this cohort of CCS. Although we did not measure markers of bone turnover, other studies have shown that IL-6 can induce osteoclastogenesis [46], increase bone resorption [47], and inhibit bone formation by osteoblasts [48]. Studies in adult men and women, before and after menopause, have shown associations between higher levels of IL-6 and osteoporosis [49-51], and baseline IL-6 levels have predicted bone loss over a 3 year period in older adults [52]. Higher levels of IL-6 have also been shown to correlate with osteoporosis and other bone deficits in patients with chronic inflammatory diseases such as rheumatoid arthritis [53] and inflammatory bowel disease [54], as well as patients who underwent HCT [55-59]. The current study is the first to report such an association in CCS who have not received HCT.

A limitation of this study is that the bone density data were collected by DXA. DXA provides a 2-dimensional image that can result in a falsely decreased BMD in children who are small for their age simply due to small bone size [60-63]. A potential example of this in our study was the finding that mean BMD Z-scores were significantly different between CCS and healthy siblings before adjustment for height SDS, but not significantly different after adjusting for height SDS. However, CCS were on average shorter than the healthy siblings and so it is impossible to distinguish by DXA measurements whether the influence of height SDS is due to DXA underestimating BMD in shorter individuals or simply that height SDS is acting as an indicator of being in the CCS group. The mean height in our CCS cohort was normal (0.1 ± 01 SDS) therefore we would not expect a significant underestimation of BMD by DXA. DXA is also limited in that it cannot distinguish between cortical and trabecular bone or estimate bone strength. Future studies of bone health in CCS should consider performing peripheral quantitative computer tomography (pQCT) scans to limit the impact of short stature on BMD and evaluate potential variation in impact on cortical versus trabecular bone. DXA is clinically useful because of low radiation exposure, short time required for scanning, and ample amount of scientific literature in pediatric populations. Importantly, studies have shown that not only low BMD Z-score, but any decrease in BMD Z-score predisposes children to an increased risk of fractures [33,34]. An additional limitation is that markers of bone turnover and vitamin D levels were not measured; vitamin D intake was used as a surrogate marker for 25-OH vitamin D level. Finally, in this cross-sectional study it is not possible to make any inference on causality. Despite these limitations, the study documents the presence of mild bone deficits in childhood cancer survivors (even before they reach adulthood) and identifies potentially modifiable factors associated with bone deficits.

## Conclusions

In conclusion, this is the first study comparing bone health measures between CCS and a sibling control group during childhood. We found that CCS had lower lumbar spine and total body BMD than the controls, and that although reported activity levels were no different between groups, CCS had lower lean body mass than the controls. Within the CCS group, the results show that lower lean body mass, lower levels of physical activity, higher daily television viewing time, higher IL-6 levels, hypogonadism and exposure to radiation or steroids increased the likelihood of having a BMD Z-score ≤ -1. In addition, contrary to what we expected, treatment before puberty and length of time since treatment did not influence risk of having bone deficits. A BMD Z-score ≤ -1 is expected to increase both the current and lifetime risk of fracture. Although we have not shown causality between any of these measures and bone health, prospective data in other populations support the bone health benefits of increasing lean body mass through activity and resistance exercises. Finally, our finding of the association of IL-6 with measures of bone health identifies a novel potential treatment target to prevent osteoporosis in CCS and requires prospective studies to determine what if any impact modification of this inflammatory variable will have on bone health in CCS.

## Competing interests

The authors declare that they have no competing interests.

## Authors' contributions

LEP and AP conceived of the study, participated in its design and interpretation of data, and helped to draft the manuscript. WL and PG performed the statistical analysis. ACD, ARS, WL, PG, LMS, JLP, DRD, KSB and JS participated in analysis and interpretation of data and revising the manuscript critically for important intellectual content. All authors read and approved the final manuscript.

## Pre-publication history

The pre-publication history for this paper can be accessed here:

http://www.biomedcentral.com/1471-2431/12/40/prepub
